# Potential molecular mechanisms of overgrazing-induced dwarfism in sheepgrass (*Leymus chinensis*) analyzed using proteomic data

**DOI:** 10.1186/s12870-018-1304-7

**Published:** 2018-05-08

**Authors:** Weibo Ren, Jihong Xie, Xiangyang Hou, Xiliang Li, Huiqin Guo, Ningning Hu, Lingqi Kong, Jize Zhang, Chun Chang, Zinian Wu

**Affiliations:** 1 Institute of Grassland Research, Chinese Academy of Agriculture Sciences, No.120 East Wulanchabu Road, Hohhot, 010010 Inner Mongolia China; 2 Faculty of life sciences, Inner Mongolia Agriculture University, Hohhot, 010018 Inner Mongolia China

**Keywords:** Sheepgrass, Dwarf, Overgrazing, Differentially expressed protein, Network

## Abstract

**Background:**

This study was designed to reveal potential molecular mechanisms of long-term overgrazing-induced dwarfism in sheepgrass (*Leymus chinensis*).

**Methods:**

An electrospray ionisation mass spectrometry system was used to generate proteomic data of dwarf sheepgrass from a long-term overgrazed rangeland and normal sheepgrass from a long-term enclosed rangeland. Differentially expressed proteins (DEPs) between dwarf and normal sheepgrass were identified, after which their potential functions and interactions with each other were predicted. The expression of key DEPs was confirmed by high-performance liquid chromatography mass spectrometry (HPLC–MS) using a multiple reaction monitoring method.

**Results:**

Compared with normal sheepgrass, a total of 51 upregulated and 53 downregulated proteins were identified in dwarf sheepgrass. The amino acids biosynthesis pathway was differentially enriched between the two conditions presenting DEPs, such as SAT5_ARATH and DAPA_MAIZE. The protein–protein interaction (PPI) network revealed a possible interaction between RPOB2_LEPTE, A0A023H9M8_9STRA, ATPB_DIOEL, RBL_AMOTI and DNAK_GRATL. Four modules were also extracted from the PPI network. The HPLC–MS analysis confirmed the upregulation and downregulation of ATPB_DIOEL and DNAK_GRATL, respectively in dwarf samples compared with in the controls.

**Conclusions:**

The upregulated ATPB_DIOEL and downregulated DNAK_GRATL as well as proteins that interact with them, such as RPOB2_LEPTE, A0A023H9M8_9STRA and RBL_AMOTI, may be associated with the long-term overgrazing-induced dwarfism in sheepgrass.

**Electronic supplementary material:**

The online version of this article (10.1186/s12870-018-1304-7) contains supplementary material, which is available to authorized users.

## Background

Sheepgrass [*Leymus chinensis* (Trin.) Tzvel.] is a rhizomatous perennial C3 species dominating the Eurasian Steppe grasslands [1]. It adapts well to diverse environmental conditions, such as high alkalinity and salinity, low temperature, drought and various atmospheric N deposition levels [2–5]. Grazing is the most important economic activity in grassland and is a complex process including touch, defoliation, wounding and bovine serum albumin (BSA) deposition [6]. Moderate herbivory or mowing can stimulate rapid sheepgrass growth [7], whereas long-term overgrazing generally leads to severe decreases in shoot and tiller densities, stem length, plant height and leaf length of sheepgrass, which in turn decreases the aboveground biomass and induces plant dwarfism [8–11]. Our recent study found that leaf photosynthesis were significantly decreased by grazing of the previous generation, corresponding with the dwarf phenotype of *L. chinensis* induced by grazing disturbance both in maternal plants in the field and clonal offspring in greenhouse [12]. Grassland productivity is extremely important for proper functioning of the ecosystem and supply of forage for grazing animals [13, 14]. Plant dwarfism can lead to significant declines in the aboveground biomass and productivity in grassland, causing changes in the structure and function of the grassland ecosystem through a set of cascade reactions from individual, species and population to ecosystem [15]. Therefore, it is necessary to reveal the mechanisms underlying dwarfism in sheepgrass during grazing.

Major advances have been achieved in previous studies about investigating the molecular mechanisms underlying the response of sheepgrass to grazing. After herbivory, animal saliva significantly increases tiller number, number of buds and biomass of sheepgrass, which are linked to the mobilisation of carbohydrates [7]. Based on RNA sequencing, 2002 genes were identified to be differentially expressed in sheepgrass and to respond to salivary BSA deposition during grazing, indicating that grazing affects plant recovery probably via salivary BSA [16]. Thousands of genes were also identified to be differentially expressed in *L. chinensis* after wounding and defoliation [17]. However, previous studies mainly focussed on the effects of stress caused by short-term or instantaneous grazing on sheepgrass growth at genome level, and the effect of stress caused by long-term overgrazing, particularly at the protein level, has not been studied extensively.

It is well known that proteins are vital parts of living organisms, with many functions. The term proteomics was coined in 1997 by James [18] in analogy with genomics. Proteomics is more complicated than genomics because the genome of organism is more or less constant, whereas the proteome differs from cell to cell and from time to time. Therefore, in this study we used proteomic profiling to compare the proteins expression in dwarf sheepgrass from a rangeland with a long history of overgrazing and that in normal sheepgrass from a rangeland with a long history of enclosure. Differentially expressed proteins (DEPs) between dwarf and normal sheepgrass were identified and their potential functions and interactions with each other were analysed. In addition, the expression levels of key DEPs were confirmed using high-performance liquid chromatography mass spectrometry (HPLC–MS) using the multiple reaction monitoring (MRM) method. The results may extend our understanding of proteomic changes in sheepgrass in response to long-term overgrazing and the molecular mechanisms underlying plant dwarfism.

## Methods

### Plant materials

**Fig. 1:**
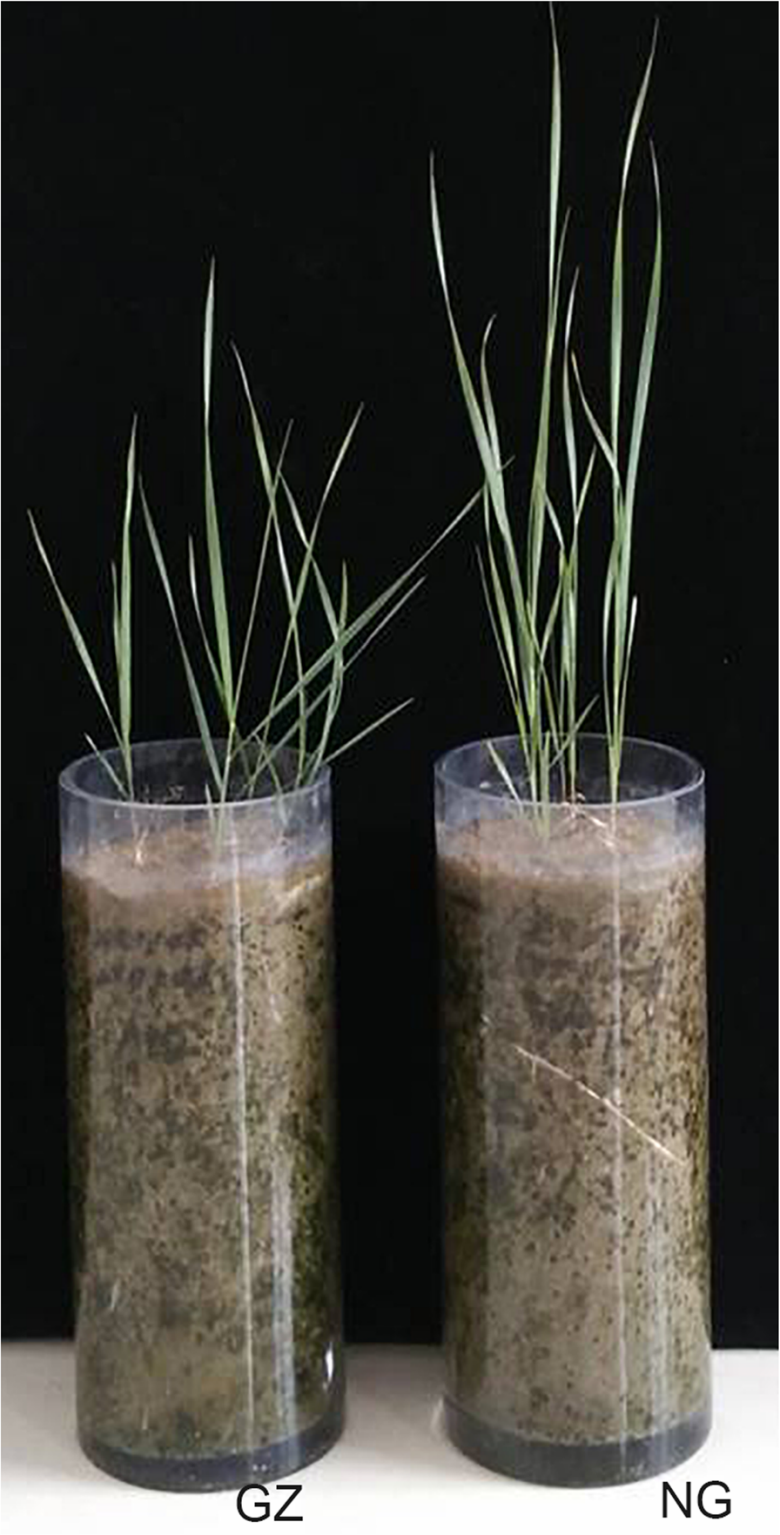
Photographs showing sheepgrass plants from long-term overgrazed rangeland (GZ) and adjacent long-term enclosed rangeland (NG)

Sheepgrass plants were sampled from a long-term overgrazed rangeland (*n* = 3, GZ group) and from an adjacent long-term enclosed rangeland that had been enclosed since 1983 for long-term ecological observation and research (*n* = 3, NG group). The two sampling sites were in fact in the same area (distance < 10 m) and were separated by a pasture fence. Photographs of the sheepgrass plants in the GZ and NG groups are depicted in Fig. 1. Both rangelands were located at the Inner Mongolia Grassland Ecosystem Research Station of the Chinese Academy of Sciences (43°38′ N, 116°42′ E). The GZ site has been grazed by approximately 600 sheep and goats throughout the year for more than 30 years at a stocking rate of approximately 3 sheep units/hectare. The plant materials used in this study were identified by Dr. Zinian Wu. The materials have been deposited in the gene bank of *Leymus chinesis* of Institute of Grassland Research, Chinese Academy of Agricultural Sciences. Additionally, the collection of plant materials has been permitted by the landowners.

After trimming the aboveground portion of sheepgrass plants to 2 cm, rhizome samples of sheepgrass were grown in an incubator at 25 °C under long-day conditions (16 h light and 8 h dark cycle) to form asexual buds. In this step, modified Hoagland’s medium (pH 6.0, adjusted with NaOH) was utilised, consisting of Ca(NO_3_)_2_∙4H_2_O (945 mg/L), KNO_3_ (506 mg/L), NH_4_NO_3_ (80 mg/L), KH_2_PO_4_ (136 mg/L), MgSO_4_∙7H_2_O (493 mg/L), H_3_BO_3_ (6.2 mg/L), MnSO_4_∙4H_2_O (22.3 mg/L), ZnSO_4_∙7H_2_O (8.6 mg/L), KI (0.83 mg/L), CuSO_4_∙5H_2_O (0.025 mg/L), CoCL∙6H_2_O (0.025 mg/L), NaMoO_4_∙2H_2_O (0.25 mg/L), FeSO_4_∙7H_2_O (13.9 mg/L) and NaFe-EDTA (18.65 mg/L). Subsequently, asexual buds were separated from the parent body and grown under the same conditions for 50 days to obtain young seedlings. The seedlings (three biological replicates for each group) were then washed with distilled water; samples were collected from leaves using sterilised scissors and tweezers. The samples were immediately frozen in liquid nitrogen and stored at − 80 °C for further analysis.

### Protein preparation and analysis

The samples were grinded into a powder in liquid nitrogen, and then the powder was immersed in 30 mL acetone containing 10% (*w*/*v*) trichloroacetic acid overnight. Following refrigerated high-speed centrifugation, the sediment was washed three times with acetone and dried. SDT-generated lysates were ultrasonicated and centrifuged. The protein level in the supernatant was quantified using the micro-bicinchoninic acid method (Pierce) [19]. Then, the proteins were separated using 12.5% sodium dodecyl sulphate polyacrylamide gel electrophoresis. Proteins were hydrolysed into peptides using trypsin [20], and the peptides were quantified at 280 nm. Each peptide sample (70 μg) was labeled using the iTRAQ Reagent-8plex Multiplex Kit (AB SCIEX, Foster City, CA, USA). The labelled peptides were separated on a reversed-phase C18 column (75 μm × 250 mm, 3 μm; Column Technology Inc., Fremont, CA, USA) in a capillary high-performance liquid chromatograph (EASY nLC1000; Proxeon, Denmark) with a linear gradient of 0–55% mobile phase B (0.1% formic acid–84% acetonitrile) in mobile phase A (0.1% formic acid aqueous solution) from 220 to 228 min, followed by a linear gradient of 55–100% mobile phase B from 229 to 240 min. Finally, the obtained fractions were analyzed on a Q-Exactive mass spectrometer (MS) (ThermoFinnigan, CA, USA) (full scan range: 300–1800 m/z, detection mode: positive ion, MS1 resolution: 70,000 at 200 m/z, automatic gain control target: 3e6, maximum IT: 20 ms, dynamic exclusion: 25.0 s, and number of scan ranges: 1). Ten fragment maps were collected in each full scan for the MS2 scan (activation type: higher energy collisional dissociation, isolation window: 2 m/z, resolution: 17,500 at 200 m/z, microscans: 1, maximum IT: 60 ms, normalised collision energy: 29 eV, and underfill ratio: 0.1%). The three biological replicates in two groups were analyzed independently.

The proteomic data are available at the EMBL-EBI Proteomics Identifications (PRIDE) database with the accession number PXD006548.

### Data preprocessing

Raw proteomic data were converted into mzXML data using ReAdW software (http://tools.proteomecenter.org/wiki/index.php?title=Software:ReAdW). mzXML data was then matched with the Swiss-Prot/TrEMBL database (release-2015_07) (http://www.uniprot.org/uniprot) [21, 22] using the PeptideProphet tool in Trans-Proteomic Pipeline software (http://www.proteomecenter.org) [23]. The matching results were obtain using pepXMLTab software. The following search parameter settings were used: peptide tolerance, ± 20 ppm and tandem mass spectrometry tolerance, 0.1 Da. A peptide was required to have at least a single assigned fragment and only unique peptides were used for protein identification. Protein with a false discovery rate (FDR) < 0.01 were considered to have high credibility.

### Functional annotation and subcellular localisation of proteins

Functional annotation was performed for the proteins with high credibility based on the Gene Ontology (GO) database (http://www.geneontology.org/) [24] and the Kyoto Encyclopedia of Genes and Genomes (KEGG) Pathway database (http://www.kegg.jp/kegg/pathway.html) [25] using the enrichment analysis tool Database for Annotation, Visualization and Integrated Discovery (DAVID) version 6.8 [26]. The subcellular localisation of the proteins was determined through Cell-PLoc 2.0, a package of web servers for predicting the subcellular localisation of proteins in various organisms [27].

### DEPs identification and functional analysis

The *t*-test method in limma package (http://www.bioconductor.org/packages/release/bioc/html/limma.html) was utilised to identify DEPs between GZ and NG groups. The *p*-value for each protein was adjusted by the Benjamini–Hochberg method [28]. Only proteins meeting the cut-off criteria of fold change > 1.2 and adjusted p-value < 0.05 were identified as DEPs. The DEPs with similar expression patterns were clustered with pheatmap software [29].

To reveal potential functions of DEPs, they were subjected to functional enrichment analysis based on the GO database, and only GO terms with *p*-value < 0.05 were considered significant. The pathway location of DEPs was determined using KEGG Mappertool (http://www.kegg.jp/kegg/mapper.html).

### Construction of protein–protein interaction (PPI) network for DEPs

Protein sequences of DEPs in fasta format were downloaded from the UniProt database (http://www.uniprot.org/) [30] and then homologous proteins of these DEPs in *Arabidopsis thaliana* were entered into the Search Tool for the Retrieval of Interacting Genes/Proteins (STRING) database (http://string-db.org/) [31], which contains known and predicted protein interactions. PPIs obtained from STRING with a combined score > 0.4 were used to construct a PPI network that was visualised using Cytoscape (http://www.cytoscape.org/) [32]. In addition, modules from the PPI network were screened using the plug-in MCODE in Cytoscape to identify potential key protein networks.

### Determination of the DEP expression using HPLC–MS

Labelled protein powder samples in the GZ (*n* = 3) and NG groups (*n* = 3) were dissolved in solution A (H_2_O:acetonitrile:formic acid = 98:2:0.1). From each sample, 1 μL was absorbed and pooled. The pooled sample was centrifuged and the supernatant was enriched in the C18 column (100 μm i.d. × 20 mm, 5 μm) and then graded in the C18 column (150 μm i.d. × 100 mm, 1.9 μm) using a gradient elution pattern. The mobile phase A was 2% acetonitrile with 98% H_2_O and 0.1% formic acid. Mobile phase B was 80% acetonitrile with 20% H_2_O and 0.1% formic acid. The flow rate was set at 0.6 μL/min.

After the gradient elution, total six samples in two groups were entered into a Q ExactiveHPLC system (Thermo Fisher Scientific, Hudson, NH, USA) equipped with an NCS3500 MS system (Dionex, Sunnyvale, CA, USA). Full MS scan with MRM mode used the following settings: scan range = 300–1400 Da, scan time = 90 min, spray voltage = 2.20 KV, capillary temperature = 320 °C, normalised collision energy = 27%, first-order scanning resolution = 120,000, AGC = 3e6 and maximum IT = 80 ms. The top 20 ions were chosen for the second scan in accordance with the following conditions: second-order scanning resolution = 150,000, AGC = 5e4 and maximum IT = 45 ms.

Finally, the generated data were analyzed using Skyline software [33] and a reference atlas database was created using the MASCOT (version: 2.1.0) [34] protein identification platform (Matrix Science, London, UK). The peptide fragments with idotp and dotp > 0.9 were considered to be credible.

## Results

### Protein identification and function annotation

After matching MS/MS data with protein databases, 23,387 peptides were identified, among which approximately 80% had 13–27 amino acids. A total of 6555 proteins were found from six samples, 1022 of which had high credibility (FDR < 0.01) (Additional file 1).

**Fig. 2:**
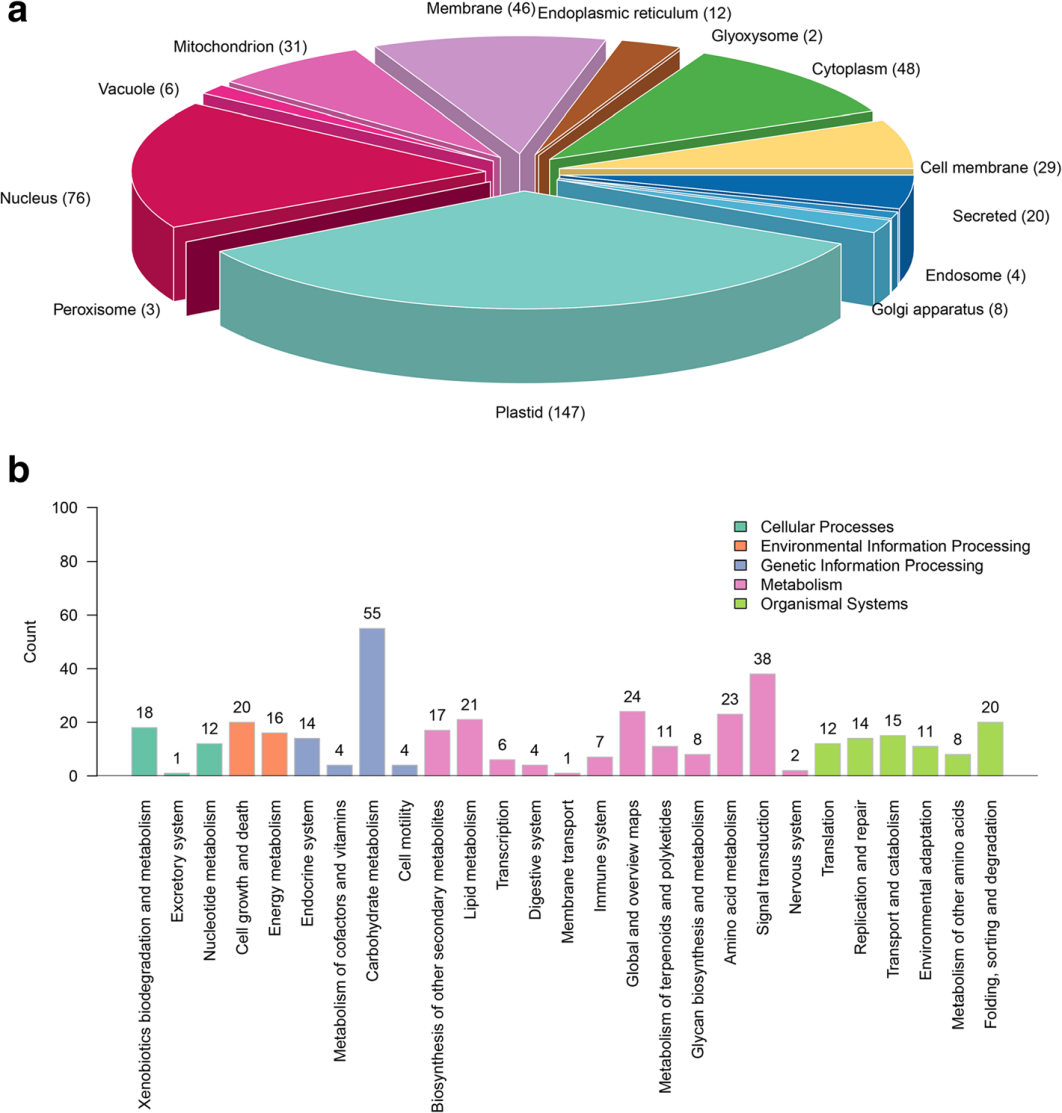
Results of subcellular localisation of the differentially expressed proteins (**a**) and the KEGG pathway annotation (**b**). KEGG, Kyoto Encyclopedia of Genes and Genomes. In **a** and **b**, the arabic numerals represent the number of proteins

The functional annotation of these 1022 proteins revealed that most of them were cytoplasmic proteins and nucleoproteins related to carbohydrate metabolism (Fig. 2a). According to the subcellular localisation analysis, 147 proteins were located in the plastid, 76 in the nucleus and 48 in the cytoplasm (Fig. 2b).

### Functional analysis of DEPs

In total, 104 proteins were differentially expressed between GZ and NG groups, with 51 being upregulated and 53 downregulated in the GZ group (Additional file 1). GO enrichment and KEGG pathway location analyses revealed that the upregulated DEPs were particularly associated with several GO terms related to metabolic pathways, such as pseudouridine synthesis (B8BQ07_THAPS), polyamine catabolic process (PAO3_ARATH) and haem biosynthetic process (J3LTE1_ORYBR), (Table 1). In addition, the downregulated DEPs were markedly associated with functions such as reductive pentose-phosphate cycle (RBL_AMOTI and A0A023HP98_9POAL), cytoplasmic translation (RS72_ARATH) and DNA replication-related DNA unwinding (GYRA_ARATH) (Table 2). The pathway location analysis mainly localised DEPs in metabolic pathways (e.g., SAT5_ARATH, PME21_ARATH and DAPA_MAIZE), such as, biosynthesis of secondary metabolites (SAT5_ARATH, DAPA_MAIZE and CAS1_ARATH), microbial metabolism in diverse environments (SAT5_ARATH, DAPA_MAIZE and DPNP1_ARATH) and biosynthesis of amino acids (SAT5_ARATH and DAPA_MAIZE) (Table 3). All results of GO and pathway enrichment analyses are shown in Additional file 1.

**Table 1:** Results of the Gene Ontology enrichment analysis of upregulated differentially expressed proteins

GO ID	GO terms	*p*-value	Proteins
GO:0042626	MF:ATPase activity, coupled to transmembrane movement of substances	0.019365668	AB21B_ARATH; NAP5_ARATH
GO:0004386	MF:helicase activity	0.037111391	RH52A_ORYSJ; R7W1Y4_AEGTA; RH12_ARATH
GO:0001522	BP:pseudouridine synthesis	0.039927405	B8BQ07_THAPS
GO:0006598	BP:polyamine catabolic process	0.039927405	PAO3_ARATH
GO:0006783	BP:heme biosynthetic process	0.039927405	J3LTE1_ORYBR
GO:0042545	BP:cell wall modification	0.039927405	PME21_ARATH
GO:0046208	BP:spermine catabolic process	0.039927405	PAO3_ARATH
GO:0005667	CC:transcription factor complex	0.043557169	V7CFX3_PHAVU
GO:0033115	CC:cyanelle thylakoid membrane	0.043557169	ATPX_CYAPA
GO:0045263	CC:proton-transporting ATP synthase complex, coupling factor F(o)	0.043557169	ATPX_CYAPA
GO:0009738	BP:abscisic acid-activated signaling pathway	0.046248498	DPNP1_ARATH; EMBP1_WHEAT

*GO* Gene Ontology, *BP* biological process, *CC* cellular component

**Table 2:** Results of the Gene Ontology enrichment analysis of downregulated differentially expressed proteins

GO ID	GO terms	*p*-value	Proteins
GO:0005484	MF:SNAP receptor activity	0.00415773	SYP51_ARATH; NPS11_ARATH
GO:0019253	BP:reductive pentose-phosphate cycle	0.010353345	RBL_AMOTI; A0A023HP98_9POAL
GO:0004497	MF:monooxygenase activity	0.02239863	RBL_AMOTI; A0A023HP98_9POAL; T5H_TAXCU
GO:0022626	CC:cytosolic ribosome	0.024251396	MCCA_ORYSJ; RS72_ARATH
GO:0031902	CC:late endosome membrane	0.024251396	SYP51_ARATH; NPS11_ARATH
GO:0003824	MF:catalytic activity	0.036656428	A0A059B3X1_EUCGR; L1JQK1_GUITH
GO:0002181	BP:cytoplasmic translation	0.043557169	RS72_ARATH
GO:0006268	BP:DNA unwinding involved in DNA replication	0.043557169	GYRA_ARATH
GO:0006414	BP:translational elongation	0.043557169	RLA2A_MAIZE
GO:0006535	BP:cysteine biosynthetic process from serine	0.043557169	SAT5_ARATH

*GO* Gene Ontology, *BP* biological process, *CC* cellular component, *SNAP* soluble N-ethylmaleimide-sensitive factor attachment protein

**Table 3:** KEGG pathways in which differentially expressed proteins are located

Pathway	Protein	Style
ko01100 Metabolic pathways	SAT5_ARATH	down
	PME21_ARATH	up
	DAPA_MAIZE	down
	CAS1_ARATH	down
	MCCA_ORYSJ	down
	D0NWK3_PHYIT	up
	DPNP1_ARATH	up
ko01110 Biosynthesis of secondary metabolites	SAT5_ARATH	down
	DAPA_MAIZE	down
	CAS1_ARATH	down
ko01120 Microbial metabolism in diverse environments	SAT5_ARATH	down
	DAPA_MAIZE	down
	DPNP1_ARATH	up
ko01230 Biosynthesis of amino acids	SAT5_ARATH	down
	DAPA_MAIZE	down
ko03010 Ribosome	RLA2A_MAIZE	down
	RS72_ARATH	down

*KEGG* Kyoto Encyclopedia of Genes and Genomes

### PPI network

**Fig. 3:**
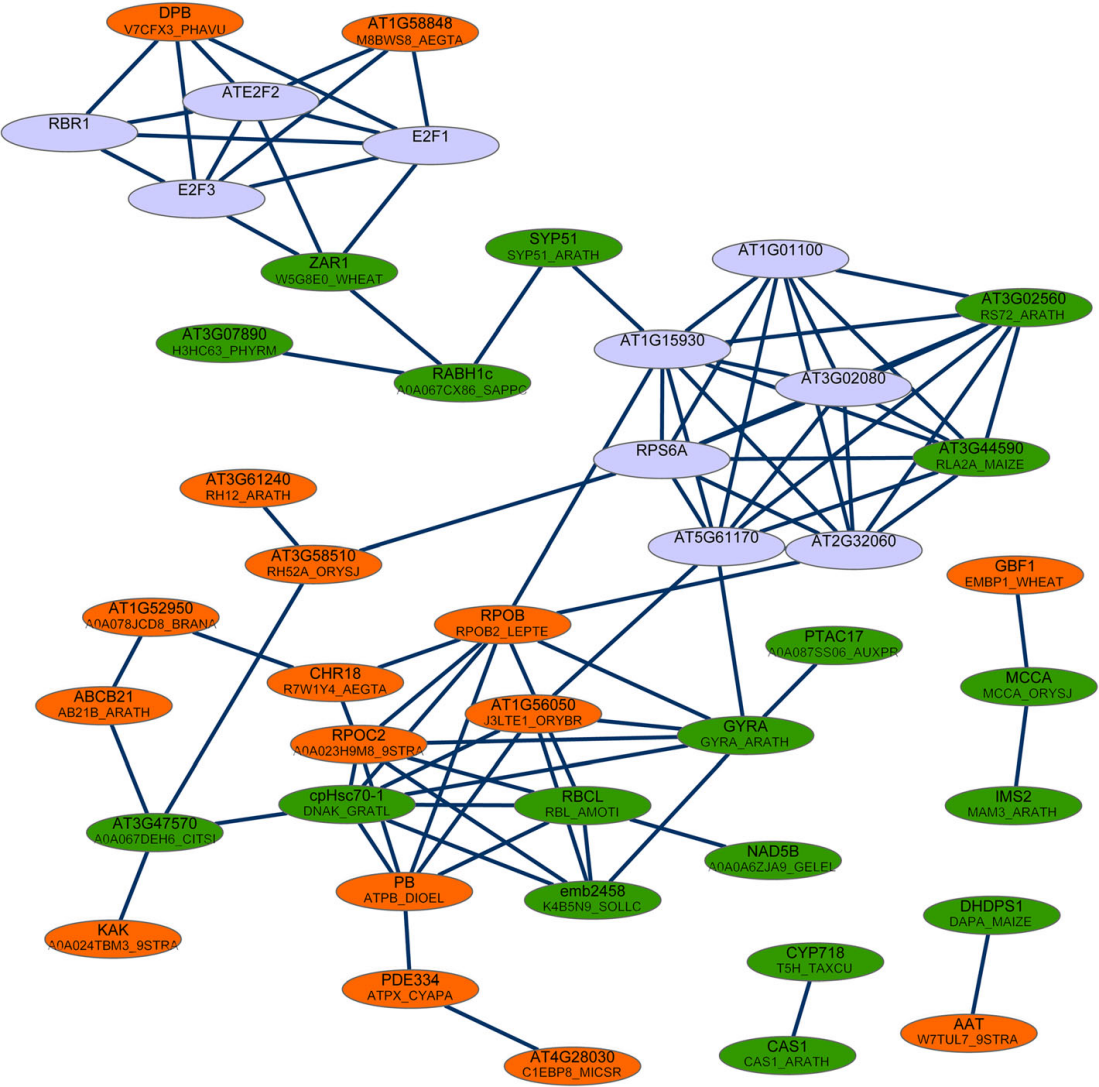
The protein–protein interaction network of the differentially expressed proteins. The orange nodes represent the upregulated proteins in dwarf sheepgrass, the green nodes represent the downregulated proteins and the purple nodes represent the proteins predicted to interact with the differentially expressed proteins

A total of 89 proteins in *A. thaliana* were homologous to DEPs and among them, 30 proteins were predicted to interact with each other (Fig. 3). The PPI network revealed a possible interactions between the proteins of A0A023H9M8_9STRA, RPOB2_LEPTE, ATPB_DIOEL, DNAK_GRATL and RBL_AMOTI.

**Fig. 4:**
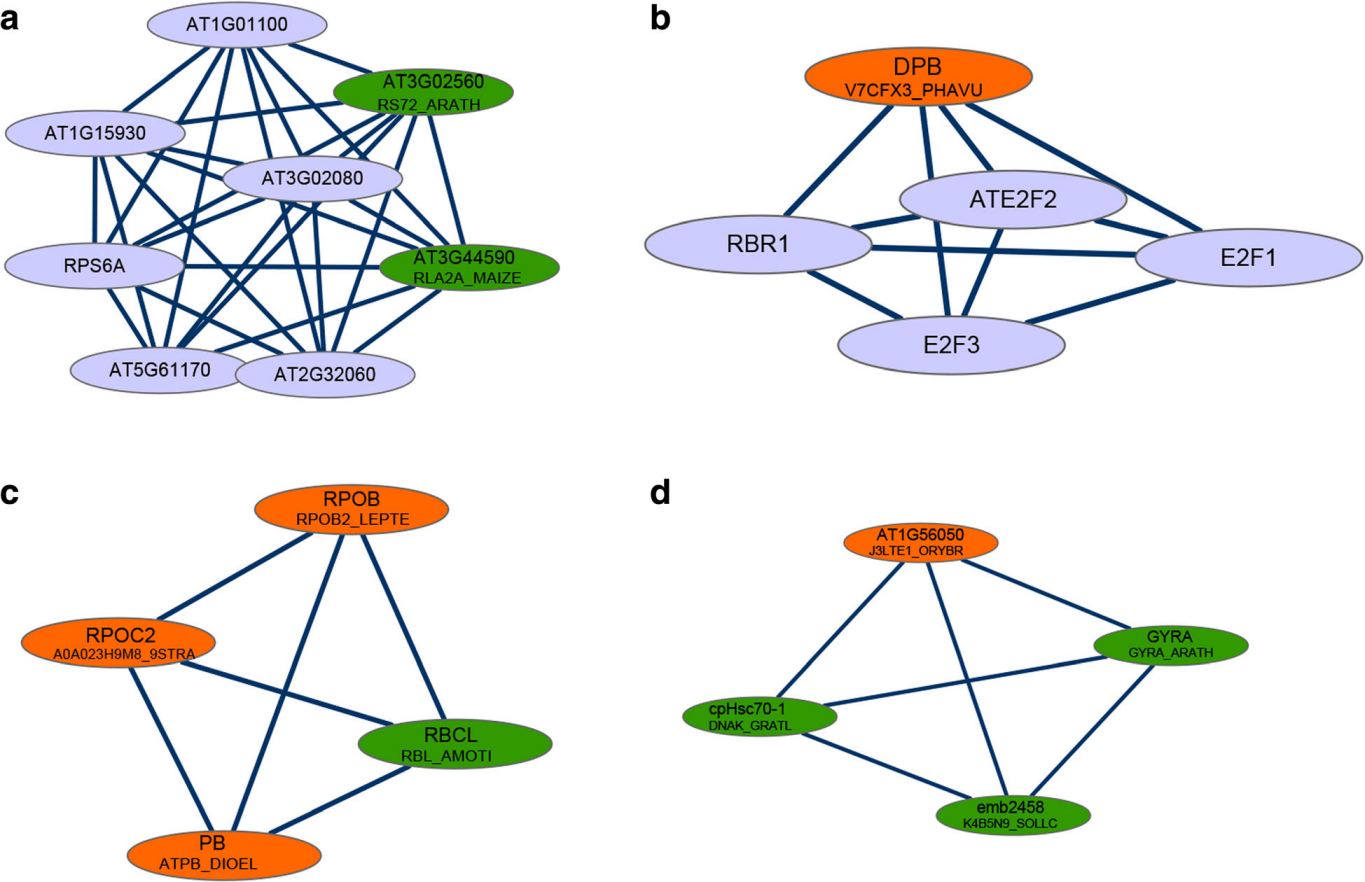
Modules that were extracted from the protein–protein interaction network. (**a**) Module 1, (**b**) Module 2, (**c**) Module 3, and (**d**) Module 4. The orange nodes represent the upregulated proteins in dwarf sheepgrass, the green nodes represent the downregulated proteins and the purple nodes represent the proteins predicted to interact with the differentially expressed proteins

Four modules were extracted from the PPI network (Fig. 4a–d). In module 3, there existed possible interactions between the proteins of RPOB2_LEPTE, A0A023H9M8_9STRA, ATPB_DIOEL and RBL_AMOTI (Fig. 4c), whereas module 4 revealed a possible interactions between J3LTE1_ORYBR, DNAK_GRATL, GYRA_ARATH and K4B5N9_SOLLC (Fig. 4d).

### Validation of DEPs expression

The HPLC–MS system with the MRM mode was used to verify proteomic data at the protein level. Information on the target peptides selected for validation is listed in Table 4. In accordance with the criteria of idotp and dotp > 0.9, three peptide fragments were credible, including two fragments of ATPB_DIOEL and one of DNAK_GRATL. ATPB_DIOEL were confirmed to be upregulated and DNAK_GRATL was confirmed to be downregulated in the GZ group (Table 5). These findings were consistent with the results of proteomic data in the present study.

**Table 4:** The information of target peptides selected for validation

Protein	Sequence of peptide fragment	Modification	Peptide-Spectrum Matches	Missing cut site	Areas: 250 ng Sample	Areas: 500 ng Sample	Areas: 1μg Sample	Ions score	Charge	m/z [Da]	Retention time [min]
A0A023H9M8_9STRA	INQDLIK		1	0		4.20E + 06		21.03	2	422.2516	58.14266
	LSLTEK		1	0			6.00E + 07	17.61	2	345.7041	16.43223
ATPB_DIOEL	FVQAGSEVSALLGR		41	0	3.60E + 09	6.20E + 09	7.70E + 09	80.86	3	478.5956	58.26444
	VVDLLAPYR		18	0	2.80E + 09	4.40E + 09	6.40E + 09	65.14	2	523.3051	53.76362
	IGLFGGAGVGK		11	0	3.30E + 09	5.10E + 09	7.30E + 09	50.98	2	488.2842	46.33776
RPOB2_LEPTE	LVAAILK		3	0	1.10E + 07	1.80E + 07	2.70E + 07	21.84	2	364.2568	43.77067
	LLINR		4	0	1.00E + 08	1.40E + 08	1.90E + 07	34.12	2	314.71	24.25685
M8BWS8_AEGTA	SFLICDK	1 × Carbamidomethyl [C5]	1	0			3.20E + 07	13.1	2	441.7259	48.12331
	LQYIR		2	0		2.50E + 07	7.70E + 07	19.66	2	346.7074	25.2921
	GGFLLLK		5	0				15.72	2	374.2409	52.32173
RH52A_ORYSJ	ALVLAPTR		4	0	4.30E + 07	9.30E + 07	1.10E + 08	30.71	2	420.7678	33.04348
	MLDMGFEPQIR		3	0	7.00E + 07	1.10E + 08	1.50E + 08	38.48	2	668.822	57.49355
DNAK_GRATL	LVGQIAK		3	0	2.50E + 08	4.80E + 08	5.10E + 08	26.12	2	364.7362	20.16307
	TTPSVVAYTK		2	0	1.50E + 08		2.50E + 08	62.94	2	533.7919	27.44009
RBL_AMOTI	ACYECLR	2 × Carbamidomethyl [C2; C5]	12	0	9.40E + 09	2.10E + 10	2.70E + 10	40.55	2	486.2065	24.70922
	GGLDFTK		18	0	6.80E + 09	1.10E + 10	1.20E + 10	48.64	2	369.1945	29.39921
	VALEACVQAR	1 × Carbamidomethyl [C6]	248	0	8.30E + 09	1.40E + 10	1.20E + 10	73.99	2	558.7944	80.73275
GYRA_ARATH	LSSSLLR		2	0		3.60E + 07	3.40E + 07	34.37	2	388.2366	23.16113
	IAELVENK		1	0		2.00E + 07		19.47	2	458.2661	28.9538
AB21B_ARATH	MILEK		1	0	2.10E + 07			25.58	2	317.1849	25.69967
	GDIELR		1	0				18.2	2	351.6919	20.34719
RH12_ARATH	ILDLTK		3	0	4.40E + 08	6.20E + 08		35.21	2	351.7226	35.33296
	VELLAK		6	0	6.10E + 07	8.80E + 07	1.30E + 08	26.94	2	336.7175	28.20831

**Table 5:** Results of protein expressions validated by high performance liquid chromatography-mass spectrum

Protein	Sequence of peptide fragment	Parent ion	Daughter ion	Ratio (GZ group/NG group)	Ratio (GZ/NG)
ATPB_DIOEL	IGLFGGAGVGK	488.2847	862.4781	1.093358789	1.115672
			488.2827	1.110895857	
			545.3042	1.09431197	
			692.3726	1.098179875	
	VVDLLAPYR	523.3057	619.3562	1.131781561	
			732.4403	1.136628677	
			847.4672	1.130923575	
			946.5356	1.129295611	
Q6B8V2	TTPSVVAYTK	533.7926	581.3293	0.838309744	0.85
			680.3978	0.852191558	
			767.4298	0.855547494	
			864.4825	0.844801621	

## Discussion

In this study, a total of 1022 proteins with high credibility were identified from dwarf sheepgrass from the long-term overgrazed rangeland and normal sheepgrass from the long-term enclosed rangeland. Among them, 51 upregulated and 53 downregulated proteins were identified in the dwarf samples. The PPI network revealed a possible interaction between the proteins RPOB2_LEPTE, A0A023H9M8_9STRA, ATPB_DIOEL, RBL_AMOTI and DNAK_GRATL. The HPLC–MS analysis confirmed that ATPB_DIOEL was upregulated and DNAK_GRATL was downregulated in the dwarf samples.

ATPB_DIOEL is a ATP synthase subunit beta in the chloroplast. The ATP synthase complex catalyses ATP synthesis in photosynthesis, a process termed as photosynthetic phosphorylation [35]. A previous study has revealed that the activation state of ATP synthase can limit leaf-level photosynthesis [36]. In this study, the expression level of ATPB_DIOEL was confirmed to be upregulated in the dwarf sheepgrass from the long-term overgrazed rangeland. This indicated that long-term overgrazing may upregulate the expression of ATPB_DIOEL, promoting the formation of ATP synthase and thereby, limiting the photosynthesis of sheepgrass and restricting plant growth.

The expression level of DNAK_GRATL, one of the proteins predicted to interact with ATPB_DIOEL, was confirmed to be downregulated in dwarf sheepgrass, and it was heat shock protein 70 (hsp70). hsp70s can assist in a wide range of protein-folding processes in almost all cellular compartments. hsp70s have critical functions in preventing aggregation and assisting protein refolding under normal and stress conditions [37]. hsp70s are essential for plant development and hsp70 mutant plants exhibit growth retardation [38]. Therefore, in this study, decreased expression of DNAK_GRATL may be associated with long-term overgrazing-induced dwarfism in sheepgrass.

Downregulated expression of RBL_AMOTI, another protein that was predicted to interact with ATPB_DIOEL, was predicted to be associated with reductive pentose-phosphate cycle. RBL_AMOTI is the large chain of ribulose bisphosphate carboxylase (RubisCO), which is also a chloroplast enzyme and participates in the reductive pentose-phosphate cycle [39]. RubisCO catalyses the carboxylation of D-ribulose-1,5-bisphosphate, which is the primary event in both carbon dioxide fixation and pentose substrate oxidative fragmentation in the photorespiration process [40, 41]. Therefore, RubisCO can support photosynthesis and plant growth [42, 43]. In this study, the expression level of RBL_AMOTI was reduced in dwarf sheepgrass from the long-term overgrazed rangeland. Although there is no other evidence to prove the association of RubisCO with overgrazing or plant dwarfism, we speculated that the expression level of RBL_AMOTI was decreased because of long-term overgrazing, thereby reducing RubisCO synthesis and then limiting photosynthesis and slowing down sheepgrass growth.

In addition, RPOB2_LEPTE and A0A023H9M8_9STRA interacted with ATPB_DIOEL as well as DNAK_GRATL and RBL_AMOTI as shown in the PPI network. RPOB2_LEPTE is the subunit beta C-terminal section of DNA-directed RNA polymerase that catalyses the transcription of DNA into RNA [44]. A0A023H9M8_9STRA is encoded by the chloroplast gene rpoC2, which also encodes the beta subunit of RNA polymerase [45]. Currently, there is no evidence to support the associations of these two proteins with plant growth or overgrazing. We speculated that RPOB2_LEPTE and A0A023H9M8_9STRA are involved in long-term overgrazing-induced dwarfism in sheepgrass through their interactions with other proteins, such as ATPB_DIOEL, DNAK_GRATL and RBL_AMOTI.

Furthermore, the present study showed that metabolic pathways such as the biosynthesis of secondary metabolites and the biosynthesis of amino acids were particularly associated with a series of DEPs, such as SAT5_ARATH and DAPA_MAIZE if applicable. SAT5_ARATH (serine acetyltransferase 5) is a key enzyme in cysteine biosynthesis during sulphur assimilation in higher plants [46]. Sulphur is required for the growth of all organisms, and inorganic sulphate in soil can be assimilated by plants to synthesise sulphur-containing amino acids, such as cysteine and methionine, to support plant growth [47]. However, we found that the expression of SAT5_ARATH was downregulated in dwarf sheepgrass. We speculated that long-term overgrazing decreased the expression of SAT5_ARATH, thereby inhibiting sheepgrass growth. DAPA_MAIZE [4-hydroxy-tetrahydrodipicolinate (HTPA) synthase] is a homotetrameric enzyme of lysine biosynthesis that catalyses the condensation of (S)-aspartate-beta-semialdehyde [(S)-ASA] and pyruvate to HTPA [48]. A lysine-rich arabinogalactan protein AtAGP19 functions in multiple processes during plant growth and development, including cell division and expansion, leaf development and reproduction [49]. Protein lysine acetylation plays regulatory roles in the photosynthesis process and Calvin cycle in plants [50–52]. These results indicate the critical role of lysine in plant growth. In the current study, the expression level of DAPA_MAIZE was decreased in dwarf sheepgrass. Therefore, long-term overgrazing reduced the expression level of DAPA_MAIZE in sheepgrass, which may suppress photosynthesis, leading to the exhibition of dwarfism.

A previous study of Huang et al. [16] employed next-generation sequencing technology to characterize de novo the transcriptome of sheepgrass after defoliation and grazing treatments and to identify differentially expressed genes responding to grazing and BSA deposition. Enrichment analysis of 2002 differentially expressed genes revealed that the effects of grazing and BSA deposition involved cell oxidative changes and apoptosis. However, our present study did not enrich functions associated with cell oxidative changes and apoptosis. Instead, we obtianed several proteins that may be related to photosynthesis, which was in consistent with our recent study as we mentioned above [12].

Despite the aforementioned results, a main limitation of the present sutdy is that the protein interaction and their functions were not confirmed by experiments. Therefore, in future studies, we will experimentally confirm the expression of the proteins (e.g. RPOB2_LEPTE, A0A023H9M8_9STRA, ATPB_DIOEL, RBL_AMOTI and DNAK_GRATL) and their interactions in dwarf sheepgrass as well as the functions of SAT5_ARATH and DAPA_MAIZE. We plan to further investigate the associations of these proteins with dwarfism in sheepgrass.

## Conclusions

In conclusion, based on ESI-MS data, 104 proteins were identified to be differentially expressed between dwarf sheepgrass from the long-term overgrazed rangeland and normal sheepgrass from the long-term enclosed rangeland. HPLC–MS analysis confirmed that the expression levels of ATPB_DIOEL were upregulated in dwarf sheepgrass and those of DNAK_GRATL were downregulated. These two proteins and the proteins with which they interact, as shown in the PPI network, such as RPOB2_LEPTE, A0A023H9M8_9STRA and RBL_AMOTI, may be associated with the long-term overgrazing-induced dwarfism in sheepgrass. The downregulated expression levels of SAT5_ARATH and DAPA_MAIZE may also play key roles in this dwarfism probably via amino acid synthesis. These results provide novel information for further experimental studies and contribute to a better understanding of the molecular mechanisms underlying dwarfism in sheepgrass.

## Additional file

Additional file 1: The identified 1022 proteins that had high credibility (FDR < 0.01); 104 differentially expressed proteins between long-term overgrazed rangeland (GZ) and adjacent long-term enclosed rangeland (NG) groups; all results of Gene Ontology (GO) and Kyoto Encyclopedia of Genes and Genomes (KEGG) pathway enrichment analyses. (XLS 942 kb)

## Abbreviations

BSA: Bovine serum albumin
DEP: Differentially expressed proteins
FDR: False discovery rate
GO: Gene Ontology
HPLC–MS: High-performance liquid chromatography mass spectrometry
KEGG: Kyoto Encyclopedia of Genes and Genomes
MRM: Multiple reaction monitoring
MS: Mass spectrometer
PPI: Protein–protein interaction
PRIDE: Proteomics Identifications
